# Modified Atkins diet in advanced malignancies - final results of a safety and feasibility trial within the Veterans Affairs Pittsburgh Healthcare System

**DOI:** 10.1186/s12986-016-0113-y

**Published:** 2016-08-12

**Authors:** Jocelyn L. Tan-Shalaby, Jennifer Carrick, Krystal Edinger, Dana Genovese, Andrew D. Liman, Vida A. Passero, Rashmikant B. Shah

**Affiliations:** 1 Veteran Affairs Pittsburgh Healthcare System, Pennsylvania Pittsburgh, USA; 2 University of Pittsburgh School of Medicine, Pennsylvania Pittsburgh, USA

## Abstract

**Background:**

Dysfunctional mitochondrial processes limit malignant cells ability to use energy from fatty acids and ketones. Animal studies using ketogenic diets for cancer show encouraging results. We tested the diet’s safety and feasibility in cancer patients across a broad variety of solid tumors.

**Methods:**

We recruited 17 advanced cancer patients who were not on chemotherapy. They consumed 20 to 40 g of carbohydrates daily with evaluations performed weekly until week 4, then every 4 weeks until 16 weeks. Quality of life questionnaires monitored for tolerability and compliance. Positron emission/computerized tomography was ordered at baseline, 4,8 and 16 weeks. Student t-testing evaluated differences between baseline and last visit scores for quality of life, weight, body mass index, and serum parameters. Correlations between weight loss and serum ketones, glucose, lipids and creatinine were done. Two-tailed unpaired t-testing of the mean weight loss compared responders against non-responders.

**Results:**

Eleven out of seventeen enrolled patients were evaluable. Mean age was 65+/- 11.7 years, weight 203 +/- 4.98 lbs. (92 ± 2.3 kgs.) and previous treatment failures was 1.7, +/- 0.97. All lost significant weight with hematologic, biochemical and lipid tests remaining stable. Quality of life scores slightly improved. At 4,8 and 16 weeks, six (54.5 %), five (45.4 %) and four (36 %) patients were stable or improved. We observed no correlations between serum glucose, ketones or lipids. Clinical response did not correlate with ketosis or glycemia. Responders (stable disease or partial responders) lost statistically more weight than non-responders. Dietary compliance was difficult. Only three patients continued dieting past 16 weeks. Out of these, two patients developed brain metastases and were on steroids. They survived 80 and 116 weeks respectively. The third patient underwent residual tumor resection and has no disease at 131 weeks.

**Conclusions:**

Modified Atkins diets are safe and feasible in advanced cancer. Quality of life was preserved. Patients who lost at least 10 % of their body weight responded the best. Steroid intake affected optimal ketone and glucose levels. Despite this, survival improved in some melanoma and lung cancer patients. Further studies are recommended.

**Trial registration:**

Clinicaltrials.gov NCT01716468. Registered on September 18, 2012

## Background

Emerging evidence indicates that ketogenic diets can have therapeutic potential for a broad range of cancers in preclinical animal models and in patients [1–6]. A ketogenic diet is a high-fat low carbohydrate diet that was developed originally to treat refractory epilepsy [7]. KDs create a metabolic state similar to that seen in water-only fasting, where blood glucose levels are reduced and blood ketone bodies (D-β-hydroxybutyrate and acetoacetate) are elevated [7, 8]. Warburg first showed that aerobic fermentation (lactate production in the presence of oxygen) was a hallmark feature of many cancers [9]. This unusual metabolism of tumor cells later became known as the Warburg effect [10].

Aerobic fermentation was considered a compensatory response to an underlying deficiency in oxidative phosphorylation that could arise from any number of deficiencies in the structure, function or number of mitochondria [11]. Consequently, many tumor cells become addicted to glucose for survival and growth [8, 12, 13]. KDs could therefore put tumor cells under metabolic stress in lowering the availability of glucose and in elevating blood ketone bodies that require functional mitochondria for metabolism [13, 14].

Most normal cells of the body readily metabolize ketone bodies for energy especially when glucose levels become reduced. Indeed, ketone bodies are considered a super fuel since they enhance the ΔG of Adenosine triphosphate ATP hydrolysis while reducing production of reactive oxygen species (ROS) through the co-enzyme Q couple [15]. In contrast to normal cells, the glucose-dependent tumor cells cannot use ketone bodies effectively for energy [16]. Ketone bodies can increase ROS in tumor cells, which can be lethal to these cells [2]. Hence, diets that can target glucose availability while elevating circulating ketone bodies can be a non-toxic therapeutic strategy for managing those tumor cells that express the Warburg effect.

The present study was conducted to evaluate the safety and tolerability of a modified Atkins diet on subjects with advanced cancers. Our secondary objective was to determine whether the diet influenced tumor stability or progression based on changes in fluorodeoxyglucose FDG and PET/CT imaging.

## Methods

### Recruitment and eligibility

This study was reviewed and approved by our hospital’s Independent Review Board (IRB) and listed on - (https://clinicaltrials.gov/ct2/show/results/NCT01716468, Ketogenic Diet in Advanced Cancer). Patients were recruited from the Hematology Oncology outpatient clinic of the Pittsburgh Veteran Affairs Healthcare System (Pittsburgh, PA). All patients had advanced solid malignancies that were measurable and avid on FDG PET/CT imaging. None were on chemotherapy at the time of enrollment, and all had good performance status (ECOG 0–2). Exclusion criteria included cases of active gout or kidney stones, active cardiac disease, cachexia, poorly controlled diabetes, liver dysfunction (alanine/aspartate aminotransferase) ALT/AST >3 x upper limits of normal (ULN) if with liver metastasis and > 5 x ULN if no liver metastases), renal failure (serum creatinine of > 2.0 mg/dl) or known brain metastatic disease.

#### Study details

Primary endpoint was safety and feasibility. We investigated quality of life parameters and used a modified Atkins diet as the standard diet for this trial. Patients were allowed 20 to 40 g of carbohydrates per day, during a two-day screening period and were advised on grocery shopping, and menu planning. We restricted consumption of high carbohydrate foods such as cereal, bread, rice, pasta, potatoes and all fruits, but did not restrict calories, protein or fats (Table 1).

**Table 1:** Modified Atkins Diet

Food category	Allowed	Prohibited
Fruits	None	All, fresh, dried, canned.
Beverage	water, diet drinks or diet sodas, liquor, black coffee, tea, tonic water, broth	Wine, beer, milk, fruit juices, regular soda
Vegetables	green leafy vegetables,cucumbers, celery, cauliflower, brussel sprouts, broccoli, mushrooms, onions, peppers, kale, spinach, asparagus, 5 olives a day.	Carrots, Potatoes, Squash, Beans, Tomatoes, corn, peas, squash, sauerkraut
Meats and protein	Beef, pork, poultry, turkey, lamb, venison, game fowl, fish, clams, lobster, shrimp, scallops, deli meats, bacon, lunchmeat, eggs, sliceable cheese, sour cream, cream cheese, block cheese	Breaded meats or breaded fish products, soft cheeses, processed cheese products, milk, yogurt
Miscellaneous	Salt, pepper, spices, splenda, aspartame Oil, lemon juice, ghee, coconut oil, butter, full cream, nuts (small amounts), pork rinds, canola oil, real mayonnaise, oils (limit to 2 T or <4 g of carbohydrate Oil based salad dressings	Sugar, honey, molasses, sugar alcohols, juices, maple syrup, catsup, prepared salad dressings with added sugar, fructose, corn syrup, sugar free candy or chocolates fat free dressing, low fat dairy products, peanut butter, ice cream, chips, cream based dressings
Breads, pastries and cereals	None	All rice, cereals, grains, breads, flour products, popcorn, chips, pretzels, pancakes, muffins, bagels, waffles cakes, pies.

Declining performance status, worsening disease, grade 3-weight loss, or inability to maintain at least trace ketosis were criteria for removal. Eligible patients were evaluated at weeks 4, 8, 12 and 16 or until disease progression or voluntarily termination. Patients used version three of the European Organisation for Research and Treatment of Cancer Quality of Life Questionnaire-c30 (EORTC QLQ-c30). Periodic history and physical examinations included measures of height, weight and blood pressure. Blood was drawn (5 cc.) at mid-morning (between 10 AM-12 noon) during the appointed visit days, and was evaluated for a complete blood count, metabolic panel 14, (including electrolytes, uric acid, renal, liver and cholesterol profile), and for serum ketone/beta-hydroxybutyrate determinations. All blood specimens were processed in the clinical pathology laboratory (Pittsburgh VA Healthcare System) using 0.4 cc in Beckman DXC800 and DXH800 chemistry and cell hematology analyzers. Patients were monitored for adverse effects and diet tolerability. Tumors were evaluated using PET /CT for heat imaging and RECIST version 1.1 criteria for size.

### PET/CT scanning technique

All patients with fasting blood glucose levels from 60 to 180 mg/dl were included in the study group. The patients received between 10–15 mCi of FDG intravenously, (~0.15 mCi/kg BW). An in-vivo incubation time of 60 min was allowed for optimal FDG accumulation to be seen in the tumor’s hypermetabolic areas. CT-PET imaging was performed on a Philips-Gemini TF PET scanner. Time-of-Flight 3-D acquisition involved: 50 % bed position overlap with acquisition time of 1.75 min to 2.5 min per bed position. Low dose CT scans and PET scans were obtained with standard established protocol parameters from skull base to thigh level and/or whole body as needed. Acquired CT and PET images were processed to generate 3-plane images, MIP images, and co-registered series. The studies were interpreted and compared to prior evaluations using a Philips EBW-PET workstation. Tumor size was evaluated in two dimensions on axial images. Standardized uptake values (SUV) were recorded for each area and were compared to the same area in subsequent studies. Liver SUV near the falciform ligament was used as internal control.

### Statistical and graphical analysis

The EORTC QLQ-c30 was used to assess quality of life (QOL) scores. Scores were compiled and a line graph was created depicting QOL over specific time points (baseline, and at weeks 4, 8 and 16) Fig. 2. The student *t*-test was used to evaluate the significance of differences between baseline scores and last visit scores for quality of life QOL, body weights, and body mass index (BMI). Comparisons between baseline and last visit values were also evaluated for the following blood markers: total cholesterol, the high and low density lipoproteins (HDL and LDL), triglycerides, fasting glucose, serum creatinine, white blood cell count (WBC), uric acid, albumin, and serum b-hydroxybutyrate (βOHB). Correlations were evaluated between net body weight loss and the following serum markers: serum beta-hydroxybutyrate/ketone levels, glucose, and creatinine. Correlations were also calculated between serum glucose and βOHB and between glucose versus total, HDL, LDL cholesterol and triglycerides. Two-tailed unpaired t-testing of the mean weight loss in responders versus non-responders (SD or PR) was performed using IBM SPSS Statistics Version 21 software. Testing was also performed using values of ketone βOHB and glucose levels. SUV lesion values of responders were compared with the values from non-responders.

## Results

### Recruitment and initial screening process

#### Demographics

Over 300 prospective candidates for the study were screened by phone, email, and in person. All 17 of the accepted candidates were Caucasian males, and were recruited from the Pittsburgh Veteran Affairs Hematology and Oncology outpatient clinic. The average age was 65 with a median age of 64 (42–87 years). Demographics are listed under Table 2. All patients were overweight (203 ± 4.98 lbs. or 92 ± 2.3 kgs.) and had a mean body mass index (BMI) of 29.46 ± 5. Tumor histology was diverse and included adenocarcinoma (biliary, prostate, colon, pancreas-2, lung; 6 patients), squamous cell carcinoma (lung, parotid, glottis; 4 patients), malignant melanoma (3 patients), brain tumors (glioblastoma and astrocytoma; 1 patient each), papillary thyroid carcinoma (1 patient), and hepatocellular carcinoma (1 patient). All malignancies were advanced, metastatic, and unresectable. All but three were pretreated. The mean number of previous treatment failures was 1.7. Three patients were chemo-naïve (patients 4, 15 and 17) and declined chemotherapy.

**Table 2:** Patient Demographics

	Age /Sex	Race	Body mass index	Diagnosis	Previous treat ment	Histology
1	64 Male	Caucasian	32	Bile ducts	3	cholangiocarcinoma
2	64 Male	Caucasian	27	Brain cancer	2	Grade 4 glioblastoma
3	66 Male	Caucasian	40	Prostate	2	adenocarcinoma
4	87 Male	Caucasian	37	Skin cancer	0	melanoma
5	67 Male	Caucasian	24	Renal cell	3	Clear cell
6	65 Male	Caucasian	27.5	Pancreatic	2	Adenocarcinoma
7	66 Male	Caucasian	25.5	Colon cancer	2	Adenocarcinoma
8	64 Male	Caucasian	34	Thyroid	2	Papillary
9	62 Male	Caucasian	30	Skin cancer	2	Melanoma
10	82 Male	Caucasian	31	Parotid gland/Head/neck	1	Squamous cell cancer
11	49 Male	Caucasian	27	Brain cancer	2	Astrocytoma
12	42 Male	Caucasian	29	Skin cancer	2	Melanoma
13	59 Male	Caucasian	25	Head/neck supraglottic	2	Squamous cell
14	66 Male	Caucasian	35	Liver	1	Hepatocellular carcinoma
15	54 Male	Caucasian	26	Pancreatic	0	Post lung transplant, Adenocarcinoma
16	68 Male	Caucasian	24	Lung	0	Squamous Non small cell
17	84 Male	Caucasian	23	Lung	0	Adenocarcinoma Non small cell Stage 4

Screen failures and dietary adherence: Screen failures occurred in 6/17 (35 %) patients. Patient #1 withdrew after two weeks due to personal issues. Patients # 2, #14, and #15 withdrew within one week due to deteriorating performance status unrelated to the diet. Patient #2 was an insulin dependent diabetic, but no longer required insulin after only 2 days of dieting. Patient #11 did not qualify due to absence of hyper-metabolic activity on his PET CT scan. Patient # 16 changed his mind regarding participation in the study. The remaining 11/17 patients (65 %) had a mean age of 65 ± 11.7 years, weight 203 ± 4.98 lbs. These eleven proceeded with the trial, completing between 4 to 16 weeks of dieting without significant adverse effects. Six patients (35 %) maintained the diet for 8 weeks and four patients (23 %) (# 4,9,12,17) completed 16 weeks. Of these four, only three (# 4,9 and 12) successfully dieted beyond 16 weeks. One patient (# 4) remained very active until he died at 80 weeks, another patient (#9) who also remained active died suddenly at 116 weeks. The third patient (#12) remains alive without evidence of disease at 131 weeks.

#### Adverse effects

No significant adverse effects were observed in any of treated patients. Indeed, a slight trend for improved quality of life was noted for most patients on the diet. Weight loss was observed in 8/11 patients (73 %). Hyperuricemia was observed in 7/11 patients (64 %), and hyperlipidemia in 2/11 patients (18). Pedal edema, anemia, halitosis, pruritus, hypoglycemia, hyperkalemia, hypokalemia, hypomagnesemia, fatigue and flu like symptoms were seen in 2/11 patients (18 %). Although 2 patients reported halitosis, only one was determined to be actually with ketone breath (Table 3).

**Table 3:** Adverse Effects

Adverse Effects *n =* 11	Number of patients	Percentage
Weight loss	8	73 %
Hyperuricemia	7	64 %
Hyperlipidemia	2	18 %
Pedal edema	2	18 %
Anemia	2	18 %
Halitosis	2	18 %
Pruritus	2	18 %
Hypoglycemia	2	18 %
Hyperkalemia	2	18 %
Hypokalemia	2	18 %
Hypomagnesemia	2	18 %
Flu-like symptoms/fatigue	2	18 %

#### Effect on weight, body mass index (BMI), and blood pressure

**Fig. 1:**
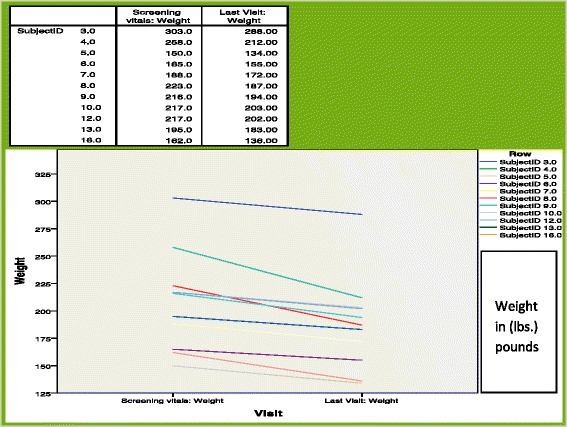
Body mass index and weight changes over time; Significant decreases in body mass index and body weight were seen when comparing baseline values versus final visit values

A mean weight loss of 3.2 lb. (1.5 kgs.) wt. loss (-1.5 %), median 3.5 SD 4.92, range 17;–11 to +6 lb. (-5 kg. to + 2.7 kg) was observed after only 2–3 days of dieting. Weight loss was consistent during the study and by week 16, subjects had lost 27 lbs. ± 13.3 or 12.3 ± 6.0 kgs. (-13 %) (Table 3, Fig. 1). Weight loss for all subjects was 16.4 ± 12.8 lbs (7.5 ± 5.8 kgs.) and highly significantly reduced compared to baseline values (*P* < 0.000108) Table 4. Three patients dieted for 16 weeks and beyond with a median percent weight loss of 10.19 % (range 6.91–17.83). BMI was also significantly decreased (*P* < 0.0004) compared to baseline values with a mean difference of 2.669 ± 1.99, (Fig. 1 : Body mass index and weight changes over time) (Table 4).

**Table 4:** Weight loss at time periods 4,8,12 and 16 weeks

	2–3 days	Week 4	Week 8	Week 12	Week 16
Mean	3.2 lbs.	13.9 lbs.	21 lbs.	23 lbs.	27 lbs.
Weight loss (lbs.)	(1.5 kg.)	(6.3 kg.)	(9.5 kg.)	(10.5 kg.)	(12.3 kg.)
% weight loss	−1.5 %	- 7 %	- 10 %	11 %	13 %
Standard deviation	±4.92	± 4.84	± 7.42	± 5.35	± 13.3

#### Hematology and blood chemistries, ketones, lipids: baseline compared to last visit values

No significant differences were seen between initial values and last visit values for biochemical and hematologic parameters (Table 5). Blood glucose values were similar at the start and at the end of the study with mean values of 92.8 ± 30 mg/dl (5.2 mM). One patient (#2) was an insulin-dependent type 2 diabetic and experienced a 100 % decrease in insulin requirements after two days on the diet and remained asymptomatic.

**Table 5:** Biochemical and hematologic parameters; Standardized Uptake Values (SUV), (Initial versus last visit)

Parameter	Mean Baseline	Standard Deviation	Mean Last Visit Value	Standard Deviation	Significance
Weight (lbs.)	210 (95 kg)	41.26 (18.7 kg)	193 (87.7 kg)	37.82	*p =* 0.0001
BMI	30.3	5.29	27.7	4.69	*p =* 0.001
Glucose (mg/dl)	89	14.8	97.1	12.07	*p =* 0.944
BHB	11.5	10.27	9.14	8.73	*p =* 0.785
BUN/Creatinine ratio	18.17	5.76	16.8	6.46	*p =* 0.108
Serum creatinine	1.14	0.23	1.09	0.21	*p =* 0.314
Systolic BP	125	10.35	127	15.59	*p =* 0.394
Diastolic BP	70	11.27	77	5.57	*p =* 0.043
Total Cholesterol	179	34.6	186	56.4	*p =* 0.819
LDL Cholesterol	125	37.6	119	40.76	*p =* 0.772
HDL Cholesterol	38.7	15.09	46.75	24.4	*p =* 0.0612
Triglycerides	114	44.45	103.58	47.70	*p =* 0.674
Uric Acid	6.34	1.42	5.92	1.73	*p =* 0.565
WBC count	7.06	4.84	7.17	3.98	*p =* 0.867
ALT	28	11.34	25	13.78	*p =* 0.193
Albumin	3.67	0.63	3.71	0.84	*p =* 0.698
SUV	2.87	1.44	2.75	1.62	*p =* 0.689

*BMI* Body Mass Index, *BHB* beta-hydroxybutyrate, *BP* blood pressure; LDL-low density lipoprotein, *HDL* high density lipoprotein

*WBC* white blood cell, *ALT* alanine transferase, *SUV* standardized uptake unit

Lipid profiles of almost all patients (94 %) remained stable throughout and did not change significantly from baseline to last visit. Total cholesterol (*p =* 0.819), LDL cholesterol (*p =* 0.772), HDL (*p =* 0.0612), triglyceride (*p =* 0.674) values at the last visit did not significantly change from baseline. Although the majority of the patients had no change in their lipid profiles, patient #12 started the trial with an elevated LDL value of 191 mg/dl, which decreased to 112 mg/dl by week 16 without using lipid lowering medication. The reverse was seen with patient # 9. This patient started with a fasting LDL of 85 mg/dl and had an LDL level of 178 by week 16, necessitating initiation of a statin medication. Blood urea nitrogen (BUN)/creatinine ratios (*p =* 0.108), serum creatinine (*p =* 0.314), albumin (*p =* 0.698), and uric acid levels (*p =* 0.565) all remained stable. White blood cell counts at baseline did not significantly differ with last visit values. Baseline PET/CT mean calculated SUV values for all lesions compared to week 4 and 8 values remained unchanged (*p =* 0.433 and *p =* 0.872 respectively). Mean SUV baseline values compared to last visit SUV also did not differ (*p* =0.77) There were no statistical difference systolic (*p =* 0.394) and diastolic (*p =* 0.43) blood pressure readings at baseline versus last visit (Table 5).

#### Glucose/ketone index

The Glucose/Ketone Index (GKI) is a ratio of the glucose (mmol) over beta-hydroxybuytrate levels (mmol) at any given time [13]. As levels of blood glucose decline, the blood levels of ketone bodies should rise, intersecting at a certain point beyond which one enters the “therapeutic zone”. This is when tumor growth is expected to slow or cease. None of the patients in this study reached GKI values predicted for therapeutic benefit against cancer (<1.0). Elevated β − OHB levels were observed in all patients within the first two days of dieting. In the first two days of dieting, 7 out of 10 patients (70 %) easily achieved a ketosis levels of 3-10 mg/dl or 0.3–1.0 mM, and only 2 were non-ketotic. Patients (# 5, 6, 7, 12) achieved βOHB levels ranging from 0.3 to 4.1 mM, while the mean βOHB level was less than 0.3 mM in the other patients. Patient 13 had some difficulty with menu planning and was removed from the trial when he progressed at 4 weeks, achieving only 2 weeks of ketosis out of 4. Once ketosis was achieved, mean ketone levels remained unchanged till the last visit compared to the first positive baseline (*p =* 0.785). After only two days of fasting the mean βOHB level rose to about 11.3 mg/dl (1.1 mM) with patients 5,6,7,8 and 12 having registered serum ketosis of over 19 mg/dl or 1.83 mM (median 24, range 19–26 mg/dl or 1.83 mMol to 2.5 mM). With the exception of patient #8, all the other patients maintained this level of ketosis for the first 4 weeks. As the study progressed, a drop in serum ketone levels was seen. The mean at week 4 was 12.08 mg/dl ± 10, and declined to 7.4 mg/dl ± 4.81, and 6.17 mg/dl ±7.99 at weeks 8 and 16, respectively. This decrease in mean βOHB was not statistically significant (*p =* 0.875) indicating generally good compliance and ability to maintain ketosis with the passage of time. Note, that when converted to millimoles, the achieved values of 7 eligible patients (63 %) ranged between 0.28 mM to 0.96 mM ketones with 4 patients (36 %) achieving values between 0.96 to 4.13 mM.

#### Correlations between weight loss and serum chemistries

Non-significant differences were seen with net weight loss (lbs.) versus βOHB ketones (*p =* 0.325, *p =* 0.992, *p =* 0.897), glucose (*p =* 0.95, *p =* 0.295, *p =* 0156), or creatinine (*p =* 0.425, *p =* 0.441, *p =* 0.377) at weeks 4, 8, and 16 (Table 6).

**Table 6:** Correlations between net weight loss and serum chemistries

Test	Week 4	Week 8	Week 16
βOHB (serum) Beta hydroxybutyrate	*p =* 0.325	*p =* 0.992	*p =* 0.897
Fasting glucose	*p =* 0.95	*p =* 0.295	*p =* 0.156
Serum creatinine	*p =* 0.425	*p =* 0.441	*p =* 0.377

#### Correlations between serum glucose and chemistries

At 4 and 8 weeks, no significant correlations were found between serum glucose versus percent (%) weight loss, ketone levels, triglycerides (TG), low density lipoprotein (LDL), high density lipoprotein (HDL), and total cholesterol (Table 7).

**Table 7:** Correlations between serum glucose and weight, ketones, and lipid parameters

Test	Week 4	Week 8
% weight loss	*p =* 0.113	*p =* 0.435
BHB ketones	*p =* 0.365	*p =* 0.89
Triglycerides	*p =* 0.169	*p =* 0.88
LDL low density lipoprotein	*p =* 0.364	*p =* 0.364
HDL high density lipoprotein	*p =* 0.557	*p =* 0.66
Total cholesterol	*p =* 0.75	p = 0.42

#### Quality of life parameters

Patients who continued on the diet for 4 weeks or more (*n =* 6) reported stable well-being and symptomatology at end of their trial with no statistically significant deterioration in quality of life scores: social (*p =* 0.179), physical (*p =* 0.225), emotional (*p =* 0.313), financial difficulties (*p =* 0.347), cognitive (*p =* 0.195) and role (*p =* 0.859) functioning. There was also no significant worsening of fatigue (*p =* 1.00), insomnia (*p =* 0.798), appetite (*p =* 0.269), constipation (*p =* 0.343), diarrhea (*p =* 0.347), or pain (*p =* 0.262).

**Fig. 2:**
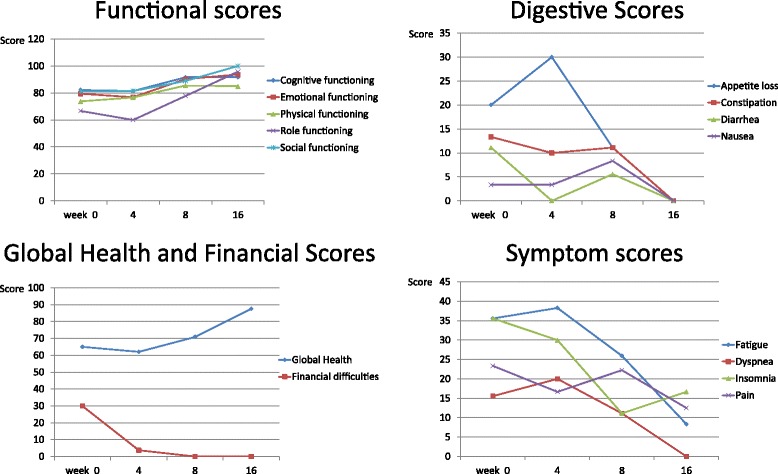
Quality of Life parameters; Scores were obtained using EORTC QLQ-c30 questionnaires. These scores were plotted against time and although there was a trend towards improvement in quality of life parameters, these differences were not statistically significant

Patients reported some improvement of cognitive functioning, dyspnea, constipation, diarrhea and insomnia. At 4 weeks there was a slight increase in the fatigue score but this also improved by 8 and 16 weeks (Fig. 2: Quality of Life parameters).

#### Responders versus non-responders

Two-tailed unpaired t testing of the mean weight loss in non-responders versus responders was performed using SPSS (Statistical Package for the Social Sciences) and Graphpad software 2015. The percentage body weight loss was significantly greater in the responders (stable disease SD or partial response PR) (*p =* 0.03, SD ± 2.28) than in the non-responders. No differences were seen between responders and non-responders in terms of serum ketone values (*p =* 0.67), fasting glucose (*p =* 0.1905), or mean calculated SUV (*p =* 0.7714).

#### Effect on the tumor

Due the low patient number, statistical analysis of the diet’s effect on tumor growth could not be performed. At the 4-week assessment, five (45 %) progressed (patients 3, 5, 6,10,13) based on RECIST 1.1 criteria and 6 (54.5 %), (patients 4,7,8,9,12,17) had stable or partially improved disease. One patient (# 5) had stable disease (decreased 3.9 %) in his target lesions at week 4 and achieved excellent ketosis (+3), but was considered progressed and had to be removed from the trial due to a newly discovered brain metastasis. Whether it was present at the beginning of the trial or was a new lesion was undetermined since he did not have baseline head scans. All the other six stable patients were allowed to diet until 8 weeks. At 8 weeks, one patient (# 7) had progressive disease, but five patients remained stable (# 4, 8, 9,12, 17) or with diminished disease. Patient # 8 withdrew voluntarily, but 4 patients (# 4, 9, 12, and 17) continued dieting until 16 weeks. At 16 weeks, four patients (36 % of total, 100 % of 8^th^ week responders) had stable disease (# 12, 16, 17) or reduced disease symptoms (# 9) at the end of their trial participation (Table 8).

**Table 8:** Diet duration, Glucose ketone index, clinical outcomes

Patient	Tumor type	Weeks on trial, weeks total on diet	Glucose/Ketone index	Outcome
1	Cholangiocarcinoma	Less than 2 weeks	NA	Dropped out of study. Menu planning difficulties.
2	Glioblastoma	4	NA	Progressive disease at 4 weeks.
3	Prostate cancer	4	14.30	Stable tumor size at 4 weeks, but PSA was rising; dropped out of study
4	Melanoma	16 on trial, continued diet post trial till 80 weeks	17 (week 4) 23 (week 8) 14 (week 16)	Stable disease at 16 weeks, mixed findings, slow progression, active until shortly before death at 80 weeks
5	Renal cell cancer	4	3.23	Progressive disease
6	Pancreas	4	2.06	Progressive disease at 4 weeks. Muscular body builder habitus. Worked out daily.
7	Colon cancer	8	8.6 (week 4) 4.3 (week 8)	Stable at 4 weeks, mixed mixed CT scan findings and rising CEA at 8 weeks.
8	Papillary thyroid	8	1.9 (week 4) 5.2 (week 8)	Stable at 8 weeks, but removed because of grade 3 weight loss; still alive 15 months later. Declined follow up bloodwork.
9	Melanoma	16 weeks on trial, continued diet post trial till 116 weeks	7.8 (week 4) 28 (week 8) 31 (week 16)	Stable disease at 16 weeks, and at 92 weeks. Active till 2 days prior to death at 116 weeks.
10	Parotid	4	18.3	Progressed at 4 weeks. Diabetic.
11	Astrocytoma	NA	NA	Disqualified due to negative PET scan
12	Melanoma	16, continued diet post trial till 100 weeks. He reported at this time that he stopped the diet at around 80 weeks.	4.5 (week 4) 12 (week 8) 80 (week 16)	Stable at 16 weeks. Underwent metastectomy of remaining axillary disease. PET/CT scan no evidence of disease at 131 weeks.
13	Head and neck	<4	12.14	Achieved ketosis in only 2 weeks out of 4. Progressed at 4 weeks.
14	Hepatoma	NA	NA	Progressed before starting diet.
15	Pancreas- post lung transplant; on multiple immunosuppresive anti-rejection drugs.	NA	NA	Very rapidly progressive disease. Progressed before starting diet.
16	Lung cancer	NA	NA	Had acute cardiac event and changed his mind before starting the trial.
17	Lung cancer	16	6.1 (week 4) 5.2 (week 8) 3.2 (week 16)	Stable at 4,8 and 16 weeks. Dieted for a week after 16, then back to standard diet. Died at 40 weeks.

All four patients who continued the diet past 16 weeks were followed in the outpatient clinic and by telephone interview. One out of the four, suffered from a case of stage IV lung cancer. The remaining three patients all had stage IV malignant melanoma, one of whom (# 12) was BRAF V600 positive.

The first 16-week patient (# 4), an 87-year-old male with diffuse multi-organ involved Stage IV melanoma, refused all forms of chemotherapy, but agreed to enroll in the modified Atkins diet trial. At 4 weeks of dieting he felt energetic and remained asymptomatic. His 4-week PET/CT scan showed mixed findings with several areas showing decreased as well as increased FDG activity. The overall clinical picture for this patient showed improved disease. By 8 and 16 weeks his disease remained stable. After 16 weeks he dieted with occasional lapses in ketosis. He developed brain metastases after 36 weeks from enrollment and was treated with steroids and gamma knife irradiation. He had difficulty maintaining ketosis but maintained his weight and active lifestyle while pain free until a few weeks before his death at age 88, marking 80 weeks from his date of enrollment.

Our second patient, (# 9), was a 62 year old male with stage IV melanoma who completed the trial at 16 weeks with stable disease. Intermittently he would be less strict with the diet and lapse out of ketosis. He had tried and progressed through ipilimumab, pembrolizumab, three rounds of transhepatic chemoembolization, two rounds of oral Temozolomide, and gamma knife radiation to his brain metastases. During the last year of his life he received no chemotherapy other than gamma knife brain irradiation plus a month of Temozolomide, and followed the ketogenic diet as his main treatment course. He was hospitalized for new onset seizures and subsequently placed on systemic steroids. Despite this he mostly remained on the ketogenic diet but because of the hyperglycemic effects of the steroids was unable to achieve the target glucose ketone index GKI. His performance status however remained excellent and he lived a total of 116 weeks from diet initiation, and nearly three years from his initial diagnosis of stage four melanoma.

Our third patient, a 42-year old male with stage IV BRAF V600 positive, Vemurafenib intolerant metastatic melanoma (patient 12), completed 16 weeks of the trial with stable disease but had persistently PET positive left axillary nodes which had not changed in size but did have some partial decrease in hypermetabolic activity while on the diet. He underwent complete surgical removal of his persistent bulky metastatic axillary nodes in July 2014 and had no adjuvant chemotherapy or radiation. He followed the modified ketogenic diet for another year after surgery and then reverted back to a standard diet at around 80 weeks. He had no evidence of disease on PET/CT scan and remained alive and well at 131 weeks.

The fourth patient, patient 17, an 84 year old with chemo-naive stage IV squamous cell lung carcinoma dieted until 16 weeks and finished the trial with stable disease while achieving excellent ketosis (+2). However, after completing the trial at week 16, he decided to revert back to low carbohydrate, non-ketotic diet. He failed to follow up for repeat imaging and died at 40 weeks.

## Discussion

This trial showed that a modified ketogenic diet was well tolerated in cancer patients with a broad variety of solid tumors. Adverse effects were minimal and none were serious. These findings are therefore consistent with other studies that treated brain cancer patients with ketogenic diets [4, 5, 11, 14, 16, 17, 18] Concerns about weight loss and malnutrition were one of the initial worries as was the possibility of deleterious effects on one’s lipid profile and quality of life. Most of our patients were either overweight (7 pts. or 42 % with BMI 25–29.9) or obese (7 or 42 % with BMI over 30) at enrolment and only 3 had normal (18 % with BMI 18.5–24.9) weight upon entering the trial. Surprisingly, weight loss did not show significant correlations with ketone levels, glucose or other hematologic or electrolyte measurements. Weight loss was seen in all patients and was significant (*p =* 0.0001). The patients who responded with either stable disease or partial responses had significantly more weight loss (*p =* 0.01) than those patients who progressed. Weight loss percentage of at least 10 % or more was seen in the responders. This weight loss was not like the cachectic weight loss that we see in cancer patients in their terminal stages because our patients maintained their quality of life scores and in some cases even improved upon them.

There was also some concern that the renal function might worsen with this diet and that diabetics might not be able to tolerate it. Renal function as well as hematologic parameters remained stable. One patient (patient 4) who began with some renal insufficiency improved his serum creatinine after 4 weeks while another patient with diabetes (patient 2) who was on insulin became insulin free within days.

Previous studies examined the effects of this diet on small patient numbers. These studies were also of short duration 4-12 weeks. We wanted to improve on this by allowing our patients to diet for a longer period of time (16 weeks). In addition we allowed long-term follow up after the 16-week period was completed. We followed four patients beyond 16 weeks and found that one (patient 17) reverted back to a standard diet within days while the other two (patients 4 and 12) continued to follow the diet without any subsequent chemotherapy or radiation, remaining in stable status for as long as 80 and 131 weeks.

Combining the diet with chemotherapy and/or radiation appears feasible. One patient (patient 9) subsequently tried a short course (one month) of oral chemotherapy and had also benefitted from invasive radiology as well as radio surgical interventions.

In the VM-M3 glioblastoma model, a low GKI is related to decreased invasion, proliferation and angiogenesis [19]. The study protocol was created prior to the introduction of the GKI and our patients did not have the benefit of frequent home glucose and ketone monitoring hence the collected in-hospital glucose and ketone readings may not be truly reflective of their actual glucose ketone indices. Discrepancies in blood draw timing and patient compliance with timing of clinic visits may have also affected some of our glucose values. Glucose ketone indices (GKI) were calculated and we found that although no patient met the targeted range of GKI, some still derived benefit, significantly outliving their estimated lifespans. One patient (patient #9) who had a good response and survived until 116 weeks, experienced persistent “ketone breath” despite achieving low βHOB ketone readings. It is possible that he achieved adequate therapeutic levels that somehow escaped detection [20, 21].

We wanted to attract a diverse population of patients, and standardize all evaluations. The small number of recruited patients limited our trial, and our recruitment was uniformly Caucasian male even though we did not exclude diverse racial types nor exclude females. Had we expanded our trial to another year’s length we perhaps could have attracted a more diverse patient sample.

One previous study had recruited patients from different institutions each with their own dietician, hence dietary instruction was not standardized across the board [4]. Blood work/scans were sent to separate laboratories and imaging facilities, possibly causing variations in data interpretations. We hoped to improve on this by recruiting all our subjects from a single institution and uniformly obtained all our testing from our inpatient hospital laboratory. Our images were read by a dedicated nuclear radiologist as well as obtained via the same dedicated PET/ CT scanner. Travel time was also an issue. Most of our patients lived a long distance from our hospital (some > 3 h away) which made recruitment difficult given the travel time and frequency of visits. Majority of the interested patients who lived locally did not qualify, as they were not US veterans, one reason for inclusion.

We chose to scan with PET/CT imaging as early as 4 weeks. Previous studies show that PET/CT scans can detect partial responses to chemotherapy as early as after one cycle [2]. In the future we feel that an 8 week PET/CT scan instead of four will be more useful as some tumors may actually be experiencing a tumor flare prior to shrinkage. We also excluded patients with brain metastases because we expected poorer performance statuses. In hindsight it could have been better to include them. Many of these patients actually do well enough to last through a short dietary course without any deleterious effects.

Not all of our patients responded to the KD. Previous work on identifying characteristics that would predict for response have studied the role of defective OXPHOS enzymes [11, 22]. The metabolic switch from oxidative phosphorylation to non-oxidative fermentation is thought to be related to upregulation of transketolase TKTL-1 protein expression which was correlated with invasive tumors [23, 24]. Identification of ketolytic and glycolytic enzyme overexpression in malignancies may help stratify those patients with low enzyme expression and accordingly should respond more favorably to the KD [11, 24].

Most of our patients were overweight and were likely to be leptin resistant and as a result have high body leptin levels. Leptin is a pro-angiogenic metabolic hormone which is secreted by adipose tissue. It sends satiety signals to the hypothalamus in response to caloric intake. Ghrelin is the opposing hormone which increases our appetite. Intake of sucrose during the fasting state results in a rise in serum leptin. However, over consumption of foods high in sucrose leads to a blunted leptin response, which leads to higher serum leptin levels [25, 26]. Unlike leptin, ghrelin secretion is suppressed by intake of both sucrose and fats. Leptin secretion is therefore probably linked to sucrose rather than fat metabolism. Increased leptin levels are associated with faster tumor growth in animal studies [27]. In humans, particularly obese individuals have higher leptin levels and correspondingly have higher rates of human cancers. A study of melanoma patients found significantly higher leptin levels in those with positive sentinel node biopsies [28]. The patients who responded with stable or improved disease lost more percentage weight, and by doing so decreased the body leptin and consequently slowed down angiogenesis.

The effect of the diet on lifespan cannot be adequately examined here due to the low number of subjects enrolled and the diverse nature of each patient’s individual tumors. However out of the four patients who did well until 16 weeks, three had a diagnosis of melanoma and all three not only went beyond 16 weeks of dieting but also significantly exceeded their expected lifespans (typically only 3 months). One remains alive and free of disease at 121 weeks. Due to variations in timing of blood draws, the reported ketone levels of these patients were not significantly elevated and none achieved the target glucose ketone index, although two did report significant halitosis which could mean that their recorded ketone levels may be underestimating their true average daily [20, 21] Whether patients with malignant melanomas have intrinsic characteristics making them more responsive to the ketogenic diet over other tumor types remains to be investigated further.

## Conclusions

The modified Atkins diet is well tolerated by patients with advanced cancer. Hematologic parameters, serum cholesterol, renal function and lipid levels remained stable. There was improvement in insulin requirements and renal function in a few patients. Quality of life measures remained stable and the diet was simple to initiate and implement. For some, the adherence to the diet remained excellent at one year and beyond. It is possible that therapeutic efficacy could be greater in future studies where patients can enter the predicted GKI zone. In this study, ideal target glucose and ketone values may have been reached but were not accurately recorded due to variations in patient compliance with blood draw timing and fasting states. The diet might help improve quality and of life and enhance tumor response to chemotherapy in cancer patients. Looking forward, the incorporation of immunohistochemical staining for ketolytic/glycolytic enzymes and biomarkers (i.e. leptin, insulin) and their role in tailoring metabolic cancer management merits further study. Notably, melanoma patients represented the three who benefitted most from this study. Future studies should investigate the modified Atkins diet MAD or the ketogenic diet KD and their potential benefits to this type of cancer.

## Abbreviation

ALT, alanine transaminase; ATP, Adenosine triphosphate; BMI, body mass index; BP, blood pressure; BUN, blood urea nitrogen; ECOG, Eastern Cooperative Group; EORTC, European Organization for Research and Treatment of Cancer; FDG, Fluorodeoxyglucose; GKI, glucose ketone index; HDL, high density lipoprotein; IRB, institutional review board, KD, ketogenic diet; LDL, low density lipoprotein; OXPHOS, oxidative phosphorylation; PET/CT, positron emission tomograpghy/computerized tomography; PR, partial response; QOL, quality of life; ROS, Reactive oxygen species; SD, stable disease; SUV, Standardized uptake units; TG, triglycerides; TKTL, transketolase; βOHB, beta hydroxybutyrate
