# Erratum to: Modified Atkins diet in advanced malignancies—final results of a safety and feasibility trial within the Veterans Affairs Pittsburgh Healthcare System

**DOI:** 10.1186/s12986-016-0120-z

**Published:** 2016-09-01

**Authors:** Jocelyn L. Tan-Shalaby, Jennifer Carrick, Krystal Edinger, Dana Genovese, Andrew D. Liman, Vida A. Passero, Rashmikant B. Shah

**Affiliations:** 1 Veteran Affairs Pittsburgh Healthcare System, Pittsburgh, Pennsylvania USA; 2 University of Pittsburgh School of Medicine, Pittsburgh, Pennsylvania USA

## Erratum

After publication of the original article [1], the author found that a word had been incorrectly used in the article body in the paragraph entitled ‘Effect on the tumor’ in the ‘Results’ section:

“The fourth patient, patient 17, an 84 year old with chemo-naive stage IV squamous cell lung carcinoma dieted until 16 weeks and finished the trial with stable disease while achieving excellent ketosis (+2).”

The word “squamous” should be “adenocarcinoma”. Therefore the correct sentence should read “The fourth patient, patient 17, an 84 year old with chemo-naive stage IV adenocarcinoma cell lung carcinoma dieted until 16 weeks and finished the trial with stable disease while achieving excellent ketosis (+2).”

## References

[1] Tan-Shalaby et al. Modified Atkins diet in advanced malignancies - final results of a safety and feasibility trial within the Veterans Affairs Pittsburgh Healthcare System*.* Nutr Metab. (2016) 13:52***.*** doi:10.1186/s12986-016-0113-y 10.1186/s12986-016-0113-y PMC4983076 27525031

